# Longitudinal neuropsychological evaluation allows diagnosis of MCI with less severe memory change

**DOI:** 10.1002/alz.70317

**Published:** 2025-05-23

**Authors:** Dona E. C. Locke, Blake Langlais, Dixie Woolston, Bryan K. Woodruff, Cynthia M. Stonnington, Leslie Baxter, Richard J. Caselli

**Affiliations:** ^1^ Division of Neuropsychology Mayo Clinic Scottsdale Arizona USA; ^2^ Division of Clinical Trials and Biostatistics Department of Quantitative Health Sciences Mayo Clinic Scottsdale Arizona USA; ^3^ Department of Neurology Mayo Clinic Scottsdale Arizona USA; ^4^ Department of Psychiatry and Psychology Mayo Clinic Scottsdale Arizona USA

**Keywords:** aging, anti‐amyloid treatment, early detection, mild cognitive impairment, neuropsychology

## Abstract

**INTRODUCTION:**

We previously demonstrated that single‐domain amnestic mild cognitive impairment (SDAm‐MCI) detected through longitudinal neuropsychological testing in our research cohort is less severe than in clinically incident symptomatic individuals. This study aims to replicate and expand this work using a National Alzheimer's Coordinating Center dataset.

**METHODS:**

Participants were classified into two groups: those enrolled with normal cognition (Normal Enrollees) and those enrolled with SDAm‐MCI (Amnestic Enrollees). Groups were compared at the time of diagnosis and on rates of cognitive decline post‐diagnosis.

**RESULTS:**

At SDAm‐MCI diagnosis, Normal Enrollees were 17% to 39% less impaired on memory tasks, after which the slope of change over time steepened compared to their pre‐symptomatic change and was steeper than in Amnestic Enrollees.

**DISCUSSION:**

Incident MCI detected through longitudinal neuropsychological testing is initially milder but may progress more steeply than in clinically established MCI. Sensitive assessment of cognitive decline is essential for capturing early changes that are more likely to be amenable to therapeutic treatment.

**Highlights:**

- Longitudinal cognitive evaluation detects symptomatic Alzheimer's disease earlier than mental status exams.
- At the time of diagnosis, converters were less impaired compared to initially symptomatic patients.
- Converters’ cognition declined more steeply than initially symptomatic patients.
- Sensitive cognitive tests are essential to capture early changes, given new disease‐modifying therapies.

## 1 BACKGROUND

In early 2023, van Dyck et al.^1^ reported that lecanemab monoclonal antibody therapy in patients with early Alzheimer's disease (AD) reduced biomarkers of amyloid and moderately slowed decline on measures of cognition and function at 18 months compared to placebo. In July 2023, lecanemab received traditional approval from the US Food and Drug Administration (FDA) for the treatment of adult patients with mild AD. Also in July 2023, Sims et al.^2^ reported similar positive results for donanemab, another monoclonal antibody treatment designed to remove brain amyloid, which received full approval from the FDA in July 2024. Dickson et al.,^3^ in an analysis of the clinical impact of donanemab in terms of “time saved” by calculating disease progression, found it was delayed by 5 months at the 18 month endpoint compared to placebo.

One important variable in determining eligibility for treatment with lecanemab and donanemab is biomarker evidence of amyloid pathology via positron emission tomography (PET) or cerebrospinal fluid (CSF) supporting AD as the etiology for cognitive impairment.^4^ However, another critical variable is the confirmation of impairment, defined as early or mild, on objective cognitive measures. In the lecanemab trial, participants were required to have memory impairment defined as performance at least one standard deviation (SD) below the age‐adjusted mean on the Wechsler Memory Scale‐IV Logical Memory II.^1^ The donanemab trial required a Mini‐Mental State Examination (MMSE) score between 20 and 28.^2^ Identification of cognitive impairment reflecting mild cognitive impairment (MCI) or mild dementia is required for clinical treatment eligibility.

In our work examining behavioral interventions for those with MCI, we found positive outcomes for self‐efficacy, quality of life, mood, memory‐related activities of daily living, daily functioning, and cognitive measurements at 12 months.^5^, ^6^, ^7^, ^8^, ^9^ Perhaps more relevant here, we found ongoing adherence to those behavioral interventions at 12 to 18 months is more likely when patients are less cognitively and functionally impaired at baseline.^10^, ^11^ Therefore, whether the outcome is time saved through slowed disease progression with pharmacotherapy or behavioral interventions, early detection of cognitive decline is essential.

Neuropsychological evaluation characterizes cognitive status and determines if subjective cognitive concerns equate to normal aging or are representative of cognitive changes in advance of normal aging. We previously reported that a diagnosis of amnestic MCI is achieved at a milder level of impairment when patients are followed longitudinally with neuropsychological evaluation compared to one‐time evaluation when subjective symptoms arise.^12^ The objective of the current project is to replicate a similar analysis within a single larger dataset and expand the analysis to include longitudinal trajectories. We hypothesized that patients with longitudinal neuropsychological assessments during a cognitively normal period prior to a single‐domain amnestic MCI (SDAm‐MCI) diagnosis would show less memory‐domain impairment at the time of diagnosis compared to patients having SDAm‐MCI diagnosed at their first neuropsychological assessment.

## 2 METHODS

### 2.1 Study data

Longitudinal data from the National Alzheimer's Coordinating Center (NACC^13^) were used for this study. NACC functions as the centralized data repository for all National Institute on Aging (NIA) Alzheimer's Disease Research Center (ADRC) programs. The program currently includes 36 centers across the United States. Each center collects data using the Uniform Data Set (UDS), which involves at least 16 different forms collected at the initial visit as well as follow‐up visits. For the purposes of this analysis, we used data from the Subject Demographics Form (A1), the Neuropsychological Battery Scores Form (C1 and C2), and Clinical Diagnosis Form (D1) collected at enrollment and follow‐up.

Participants with a diagnosis of SDAm‐MCI were first identified then isolated into two groups: (1) patients who enrolled with normal cognition, experienced their first cognitive decline diagnosis as SDAm‐MCI, and remained cognitively abnormal at subsequent visits—individuals who enrolled as normal, declined to SDAm‐MCI, and then returned to normal cognition at a later epoch were excluded (Normal Enrollees), and (2) patients who enrolled with a diagnosis of SDAm‐MCI at enrollment and remained cognitively abnormal at subsequent visits—individuals who enrolled as SDAm‐MCI and then returned to normal cognition at a later epoch were excluded (Amnestic Enrollees). For this study, time prior to and after the initial SDAm‐MCI diagnosis were considered the pre‐symptomatic and symptomatic periods, respectively. All participants must have had at least one neuropsychological assessment after the initial SDAm‐MCI diagnosis to meet study inclusion criteria, and Normal Enrollees must also have had at least one assessment prior to the initial SDAm‐MCI diagnosis visit.

Neuropsychological assessment data from the UDS C1^14^ or C2 battery^15^ were the focus of this analysis. UDS C1 was implemented in 2008 and UDS C2 in 2015. There is overlap between the two datasets but also differences, which is a significant contributor to the range in number of observations for each task. See Table S1 in supporting information for a listing of measures in each UDS battery. Although patients were assessed with a multi‐domain battery during visits, score trajectories from memory tests were the focus of this SDAm‐MCI analysis, as memory impairment is the most common initial symptom of early AD and the focus of measurement in the lecanemab trial. The focus on memory measurement is also consistent with our prior work.

### 2.2 Statistical analysis

Patient characteristics and neuropsychological battery were reported using means and SDs or counts and percentages. Characteristics and cross‐sectional neuropsychological measure data were compared between groups using *t* tests and chi‐squared tests, or Fisher exact tests as appropriate. Longitudinal neuropsychological trajectories were assessed for two scenarios: (1) estimate and compare the rates of neuropsychological decline between the pre‐symptomatic and symptomatic periods within Normal Enrollees, and (2) estimate and compare the rates of neuropsychological decline between Normal Enrollees and Amnestic Enrollees during the symptomatic period. This was achieved for both scenarios using mixed effects linear regression with random intercepts and slopes, unstructured covariance, and adjusting for patient sex and age at initial SDAm‐MCI.

Alongside the comparative group of all available Amnestic Enrollees meeting inclusion criteria, which were analyzed as our primary planned analysis, a subset of these patients was propensity score matched to Normal Enrollees (1:1) using age at initial SDAm‐MCI (i.e., one similarly aged Amnestic Enrollee for each Normal Enrollee) for a secondary analysis. Though this secondary comparative group reduces sample size of the Amnestic Enrollees, it provided an opportune means of interpretating two correspondingly aged groups’ memory decline post‐SDAm‐MCI diagnosis alongside the primary comparative group of all available Amnestic Enrollees for which age at initial diagnosis is adjusted for, using statistical modeling. Models comparing Normal Enrollee and 1:1 matched Amnestic Enrollee trajectories did not include age at initial SDAm‐MCI covariates. The first modeling scenario also used a piecewise modeling framework with a single knot (or breakpoint) imposed at the time of initial SDAm‐MCI, separating the pre‐symptomatic and symptomatic periods. Contrast analysis was used for group comparison within the respective mixed models. Graphical representations of average neuropsychological trajectories are shown after model adjustments. Follow‐up in these figures is shown centered at the time of initial SDAm‐MCI diagnosis for pre‐symptomatic versus symptomatic period comparisons among Normal Enrollees (i.e., time = 0), and starting at the time of initial SDAm‐MCI diagnosis during the symptomatic period comparing Normal and Amnestic Enrollees. Propensity score matching was carried out using the R language and environment for statistical computing (v4.3.2) and the matchit package (v4.5.5) in which logistic regression matched nearest neighbors without replacement. Statistical analyses were performed using the statistical software SAS version 9.4 (SAS Institute Inc.). *p* values < 0.05 were deemed statistically significant. *p* values and variability estimates were reported without adjustment for multiple comparisons.

**RESEARCH IN CONTEXT**

1. **Systematic review**: Literature was reviewed using traditional sources such as PubMed. Advances in treatment of Alzheimer's disease (AD) increase the importance of differentiation of normal aging from symptomatic disease as early as possible. We have previously evaluated the sensitivity of neuropsychological evaluation for this purpose; this analysis builds on those findings.
2. **Interpretation**: Our findings support the potential of brief, longitudinal neuropsychological evaluation in differentiating normal aging from early symptomatic AD before changes are seen on longitudinal mental status screening.
3. **Future directions**: There is a need to continue developing population‐based screening methods that are as sensitive as possible to cognitive change in AD to ensure that as treatments continue to develop, we can identify eligible candidates as early as possible. Access to longitudinal neuropsychological evaluation and monoclonal antibody therapies are both currently limited and as systems for dissemination of therapies evolve, or development of new early‐stage treatments, so must early detection tools.

## 3 RESULTS

### 3.1 Patient characteristics

There were 2582 patients meeting study inclusion criteria, with 21% Normal Enrollees (*n* = 552) and 79% Amnestic Enrollees (*n* = 2030). Normal Enrollees were more often female (61% vs. 48%; *p* < 0.001) and older at initial SDAm‐MCI diagnosis (82 years vs. 75; *p* < 0.001) compared to Amnestic Enrollees (Table 1). No differences were seen in ethnicity, race, or level of education. Normal Enrollees had an average of 4.3 years of pre‐symptomatic follow‐up time, and slightly less symptomatic follow‐up time compared to Amnestic Enrollees (3.5 vs. 4.1 years, respectively; *p* < 0.001). Both groups had an average of ≥ 4 visits after diagnosis, with Amnestic Enrollees having slightly more average visits (4.0 vs. 4.5 visits; *p* < 0.001). Characteristic comparisons between Normal Enrollees and the 1:1 propensity‐matched Amnestic Enrollees were comparable to the overall Amnestic Enrollees, apart from age at initial SDAm‐MCI diagnosis, and can be found in Table 2.

**TABLE 1:** Patient characteristics at initial single domain amnestic MCI diagnosis.

	Normal Enrollees (*n* = 552)	Amnestic Enrollees (*n* = 2030)	Total (*N* = 2582)	*p* value
Age at diagnosis, mean (SD)	81.6 (8.4)	75.0 (8.7)	76.4 (9.1)	<0.001^a^
Sex				<0.001^b^
Male	213 (39%)	1061 (52%)	1274 (49%)	
Female	339 (61%)	969 (48%)	1308 (51%)	
Hispanic				0.68^b^
No	523 (95%)	1912 (95%)	2435 (95%)	
Yes	28 (5.1%)	112 (5.5%)	140 (5.4%)	
Race				0.73^c^
White	477 (87%)	1762 (87.0%)	2239 (87%)	
Black or African American	56 (10.2%)	189 (9.3%)	245 (9.5%)	
Native American or Alaskan Native	1 (0.2%)	13 (0.6%)	14 (0.5%)	
Asian	12 (2.2%)	39 (1.9%)	51 (2.0%)	
Other	5 (0.9%)	22 (1.1%)	27 (1.0%)	
Education				0.46^b^
Less than HS/GRE	26 (4.7%)	107 (5.3%)	133 (5.2%)	
HS/GRE	176 (32%)	679 (34%)	855 (33.3%)	
Master's	170 (31%)	555 (28%)	725 (28%)	
Doctorate	179 (33%)	679 (34%)	858 (33.4%)	
Visits since diagnosis, mean (SD)	4.0 (2.1)	4.5 (2.6)	4.4 (2.5)	<0.001^a^
Follow‐up time since diagnosis, mean years (SD)	3.5 (2.4)	4.1 (3.0)	3.9 (2.9)	<0.001^a^

Abbreviations: HS/GRE, high school/Graduate Record Exam; MCI, mild cognitive impairment; SD, standard deviation.

^a^ *t* test.

^b^ Chi‐squared test.

^c^ Fisher exact test.

**TABLE 2:** Characteristics at time of SDAm‐MCI diagnosis by enrollment group (Normal vs. age‐matched Amnestic).

	Enrolled Normal (*N* = 552)	Enrolled Amnestic (1:1 age‐matched at time of SDAm‐MCI) (*N* = 552)	Total (*N* = 1104)	*p* value
Age at diagnosis, mean (SD)	81.6 (8.4)	81.4 (8.1)	81.5 (8.3)	0.63^a^
Sex				< 0.001^b^
Male	213 (39%)	277 (50%)	490 (44%)	
Female	339 (61%)	275 (50%)	614 (56%)	
Hispanic				0.18^b^
No	523 (95%)	532 (97%)	1055 (96%)	
Yes	28 (5.1%)	19 (3.4%)	47 (4.3%)	
Race				0.87^c^
White	477 (87%)	488 (88%)	965 (88%)	
Black or African American	56 (10%)	51 (9.2%)	107 (10%)	
Native American or Alaskan Native	1 (0.2%)	1 (0.2%)	2 (0.2%)	
Asian	12 (2.2%)	9 (1.6%)	21 (1.9%)	
Other	5 (0.9%)	3 (0.5%)	8 (0.7%)	
Education				0.14^b^
Less than HS/GRE	26 (4.7%)	38 (6.9%)	64 (5.8%)	
HS/GRE	176 (32%)	199 (36%)	375 (34%)	
Master's	170 (31%)	150 (27%)	320 (29%)	
Doctorate	179 (33%)	165 (30%)	344 (31%)	
Visits since diagnosis	4.0 (2.1)	4.3 (2.5)	4.2 (2.3)	0.036^a^
Follow‐up time since diagnosis mean years (SD)	3.5 (2.4)	3.9 (2.9)	3.7 (2.7)	0.008^a^

Abbreviations: SD, standard deviation; HS/GRE, high school/Graduate Record Exam; SDAm‐MCI, single domain amnestic mild cognitive impairment.

^a^ *t* test.

^b^ Chi‐squared test.

^c^ Fisher exact test.

### 3.2 Time of initial SDAm‐MCI diagnosis

At the time of initial diagnosis of SDAm‐MCI, Normal Enrollees showed higher memory raw scores despite older age compared to Amnestic Enrollees on nearly all memory measures (Table 3). Normal Enrollees showed 16.9% less impairment in Logical Memory Immediate Recall, 38.9% less impairment in Logical Memory Delayed Recall, 20.6% less impairment in Figure Recall, and 19.0% less impairment in Delayed Craft Story Recall. In contrast, there was no significant difference in Montreal Cognitive Assessment (MoCA) Total Raw Score (23.3 vs. 22.7; *p* = 0.062) or MMSE total score (27.2 vs. 27.0; *p* = 0.14).

**TABLE 3:** Uniform Data Set v. 3 neuropsychological battery at initial single domain amnestic MCI diagnosis.

	Normal Enrollees *n* = 552	Amnestic Enrollees *n* = 2030	Total *n* = 2582	*p* value^a^
Speed/executive				
Trails A (C1 & C2, TRAILA) mean (SD)	42.8 (20.8) *n* = 493	40.6 (19.8) *n* = 1970	41.1 (20.0) *n* = 2463	0.033^a^
Trails B (C1 & C2, TRAILB) mean (SD)	123.7 (68.1) *n* = 474	115.5 (61.6) *n* = 1934	117.1 (63.0) *n* = 2408	0.011^a^
Coding (C1, WAIS) mean (SD)	37.4 (11.3) *n* = 295	38.6 (11.6) *n* = 1470	38.4 (11.6) *n* = 1765	0.11^a^
Letter Fluency (C1 & C2, UDSVERTN) mean (SD)	27.4 (8.3) *n* = 196	27.3 (8.0) *n* = 445	27.3 (8.1) *n* = 641	0.96^a^
Visual‐spatial				
Figure copy (C1 & C2, UDSBENTC) mean (SD)	15.4 (1.3) *n* = 191	15.3 (1.4) *n* = 445	15.3 (1.4) *n* = 636	0.57^a^
Attention				
Digits Forward (C1, DIGIF) mean (SD)	8.0 (2.1) *n* = 334	8.1 (2.1) *n* = 1525	8.1 (2.1) *n* = 1859	0.42^a^
Forward Number Span (C2, DIGFORCT) mean (SD)	8.1 (2.5) *n* = 173	8.0 (2.2) *n* = 447	8.1 (2.3) *n* = 620	0.79^a^
Digits Backward (C1, DIGIB) mean (SD)	6.0 (2.0) *n* = 334	6.1 (2.0) *n* = 1524	6.0 (2.0) *n* = 1858	0.75^a^
Backward Number Span (C2, DIGBACCT) mean (SD)	7.0 (2.2) *n* = 173	6.5 (2.0) *n* = 448	6.6 (2.1) *n* = 621	0.004^a^
Language				
Animal Fluency (C1 & C2, ANIMALS) mean (SD)	16.6 (4.9) *n* = 508	16.9 (4.7) *n* = 1993	16.9 (4.8) *n* = 2501	0.16^a^
Vegetable Fluency (C1 & C2, VEG) mean (SD)	11.4 (3.8) *n* = 505	11.3 (3.7) *n* = 1970	11.3 (3.7) *n* = 2475	0.55^a^
Boston naming (C1, BOSTON) mean (SD)	25.6 (4.3) *n* = 331	25.3 (4.4) *n* = 1523	25.4 (4.4) *n* = 1854	0.37^a^
MINT naming (C2, MINTTOTS) mean SD	29.2 (3.2) *n* = 167	29.3 (2.7) *n* = 442	29.3 (2.9) *n* = 609	0.91^a^
Memory				
Logical Memory Immediate (C1, LOGIMEM) mean (SD)	9.7 (4.3) *n* = 335	8.3 (4.0) *n* = 1508	8.6 (4.1) *n* = 1843	<0.001
Logical Memory Delay (C1, MEMUNITS) mean (SD)	7.5 (4.7) *n* = 335	5.4 (4.4) *n* = 1510	5.8 (4.5) *n* = 1845	<0.001
Craft Story Immediate (C2, CRAFTURS) mean (SD)	12.0 (4.4) *n* = 174	11.4 (4.1) *n* = 441	11.6 (4.2) *n* = 615	0.09^a^
Craft Story Delay (C2, CRAFTDRE) mean (SD)	9.4 (4.9) *n* = 174	7.9 (5.1) *n* = 441	8.3 (5.1) *n* = 615	<0.001^a^
Figure Recall (C1&C2, UDSBENTD) mean (SD)	7.6 (4.0) *n* = 191	6.3 (4.0) *n* = 443	6.7 (4.0) *n* = 634	<0.001^a^
Global				
MMSE (C1, NACCMMSE), mean (SD)	27.2 (2.3) *n* = 333	27.0 (2.3) *n* = 1538	27.1 (2.3) *n* = 1871	0.14^a^
MoCA (C2, MOCATOTS), mean (SD)	23.3 (3.2) *n* = 168	22.7 (3.3) *n* = 441	22.9 (3.3) *n* = 609	0.06^a^

Abbreviations: MCI, mild cognitive impairment; MMSE, Mini‐Mental State Examination; MoCA, Montreal Cognitive Assessment; SD, standard deviation; Trails, Trail Making Test; WAIS, Wechsler Adult Intelligence Test.

^a^ *t* test.

In the secondary analysis (Table 4), matched Amnestic Enrollees showed significantly lower average scores on all memory measures at the time of initial SDAm‐MCI compared to Normal Enrollees. In this subgroup analysis, there was a significant difference on several non‐memory tasks throughout the battery as well, including Backward Digit Span, Animal Fluency, Boston Naming, MMSE total, and MoCA total, all with the matched Amnestic Enrollees showing lower scores.

**TABLE 4:** Uniform Data Set v.3 at time of SDAm‐MCI diagnosis by enrollment group (Normal vs. age‐matched Amnestic).

	Normal Enrollees (*N* = 552)	Amnestic Enrollees (1:1 age‐matched at time of SDAm‐MCI) (*N* = 552)	Total (*N* = 1104)	*p* value
Speed/Executive				
Trails A (C1 & C2, TRAILA) mean (SD	42.8 (20.8) *n* = 493	45.2 (21.6) *n* = 533	44.0 (21.3) *n* = 1026	0.07^a^
Trails B (C1 & C2, TRAILB) mean (SD)	123.7 (68.1) *n* = 474	129.4 (65.2) *n* = 512	126.7 (66.6) *n* = 986	0.18^a^
Coding (C1, WAIS) mean (SD)	37.4 (11.3) *n* = 295	35.9 (10.9) *n* = 413	36.5 (11.1) *n* = 708	0.07^a^
Letter Fluency (C1 & C2, UDSVERTN) mean (SD)	27.4 (8.3) *n* = 196	26.2 (7.3) *n* = 99	27.0 (8.0) *n* = 295	0.23^a^
Visual‐spatial				
Figure copy (C1 & C2, UDSBENTC) mean (SD)	15.4 (1.3) *n* = 191	15.1 (1.4) *n* = 100	15.3 (1.4) *n* = 291	0.14^a^
Attention				
Digits Forward (C1, DIGIF) mean (SD)	8.0 (2.1) *n* = 334	7.9 (2.0) *n* = 436	8.0 (2.1) *n* = 770	0.84^a^
Forward Number Span (C2, DIGFORCT) mean (SD)	8.1 (2.5) *n* = 173	7.9 (2.3) *n* = 100	8.0 (2.4) *n* = 273	0.49^a^
Digits Backward (C1, DIGIB) mean (SD)	6.0 (2.0) *n* = 334	5.9 (2.0) *n* = 436	6.0 (2.0) *n* = 770	0.59^a^
Backward Number Span (C2, DIGBACCT) mean (SD)	7.0 (2.2) *n* = 173	6.3 (2.0) *n* = 100	6.7 (2.2) *n* = 273	0.013^a^
Language				
Animal Fluency (C1 & C2, ANIMALS) mean (SD)	16.6 (4.9) *n* = 508	15.9 (4.5) *n* = 542	16.2 (4.7) *n* = 1050	0.014^a^
Vegetable Fluency (C1 & C2, VEG) mean (SD)	11.4 (3.8) *n* = 505	11.0 (3.8) *n* = 537	11.2 (3.8) *n* = 1042	0.12^a^
Boston naming (C1, BOSTON) mean (SD)	25.6 (4.3) *n* = 331	24.7 (4.5) *n* = 435	25.1 (4.4) *n* = 766	0.006^a^
MINT naming (C2, MINTTOTS) mean SD	29.2 (3.2) *n* = 167	29.3 (2.9) *n* = 99	29.2 (3.1) *n* = 266	0.95^a^
Memory				
Logical Memory Immediate (C1, LOGIMEM) mean (SD)	9.7 (4.3) *n* = 335	8.1 (3.9) *n* = 433	8.8 (4.2) *n* = 768	< 0.001^a^
Logical Memory Delay (C1, MEMUNITS) mean (SD)	7.5 (4.7) *n* = 335	5.3 (4.2) *n* = 443	6.3 (4.6) *n* = 768	< 0.001^a^
Craft Story Immediate (C2, CRAFTURS) mean (SD)	12.0 (4.4) *n* = 174	10.8 (4.1) *n* = 99	11.6 (4.3) *n* = 273	0.028^a^
Craft Story Delay (C2, CRAFTDRE) mean (SD)	9.4 (4.9) *n* = 174	7.5 (5.3) *n* = 99	8.7 (5.1) *n* = 273	0.003^a^
Figure Recall (C1&C2, UDSBENTD) mean (SD)	7.6 (4.0) *n* = 191	5.6 (3.6) *n* = 100	6.9 (4.0) *n* = 291	< 0.001^a^
Global				
MMSE (C1, NACCMMSE), mean (SD)	27.2 (2.3) *n* = 333	26.7 (2.4) *n* = 437	26.9 (2.3) *n* = 770	0.002^a^
MoCA (C2, MOCATOTS), mean (SD)	23.3 (3.2) *n* = 168	22.0 (3.7) *n* = 99	22.8 (3.5) *n* = 267	0.003^a^

Abbreviations: MMSE, Mini‐Mental State Examination; MoCA, Montreal Cognitive Assessment; SD, standard deviation; SDAm‐MCI, single domain amnestic mild cognitive impairment; Trails, Trail Making Test; WAIS, Wechsler Adult Intelligence Test.

^a^ *t* test.

### 3.3 Memory trajectories in Normal Enrollees before and after SDAm‐MCI diagnosis

**FIGURE 1:**
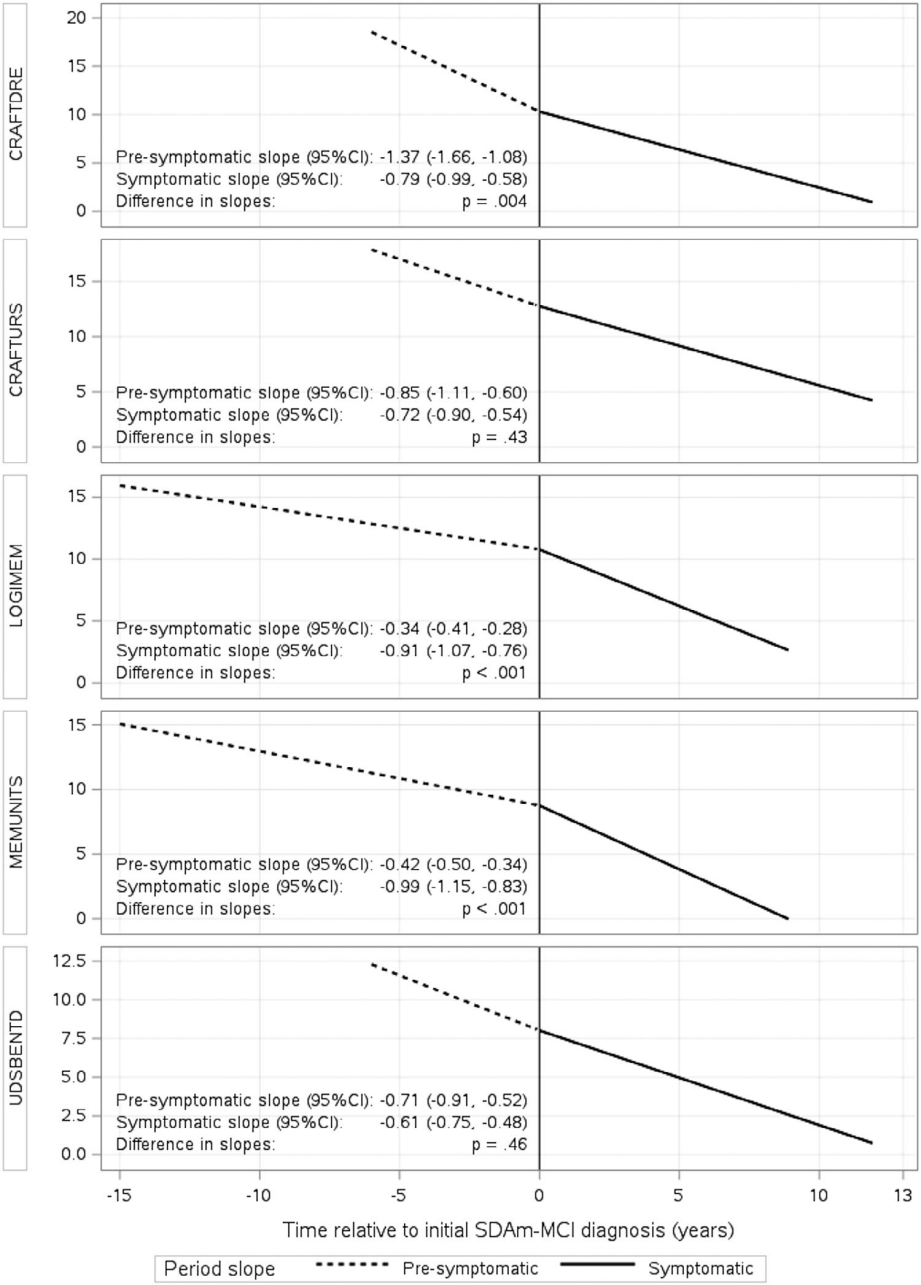
Trajectories of memory measures during the pre‐symptomatic and symptomatic periods among Normal Enrollees. Time less than 0 reflects time prior to SDAm‐MCI diagnosis (pre‐symptomatic period). Time = 0 reflects time of diagnosis. Time > 0 reflects time after diagnosis (symptomatic period). Depicted slopes represent fitted averages after adjusting for sex and age at initial SDAm‐MCI diagnosis. Linear slopes are graphically extended up to the observed minimum/maximum time from diagnosis among patients. CI, confidence interval; CRAFTDRE, Craft Story Delay; CRAFTURS, Craft Story Immediate; LOGIMEM, Logical Memory Immediate; MEMUNITS, Logical Memory Delay; UDSBENTD, Figure Recall; SDAm‐MCI, single domain amnestic mild cognitive impairment.

All memory measures showed declining trajectories during the pre‐symptomatic and symptomatic periods. Three of the five memory measures showed different rates of decline after the initial SDAm‐MCI diagnosis compared to the pre‐symptomatic period: Logical Memory Immediate Recall (*p* < 0.001) and Logical Memory Delayed Recall (*p* < 0.001) accelerated in decline, and Delayed Craft Story Recall slowed decline (*p* = 0.004). No change in rate of decline was seen in Benson Delay or Immediate Craft Story Recall (Figure 1).

### 3.4 Memory trajectories during the symptomatic period

**FIGURE 2:**
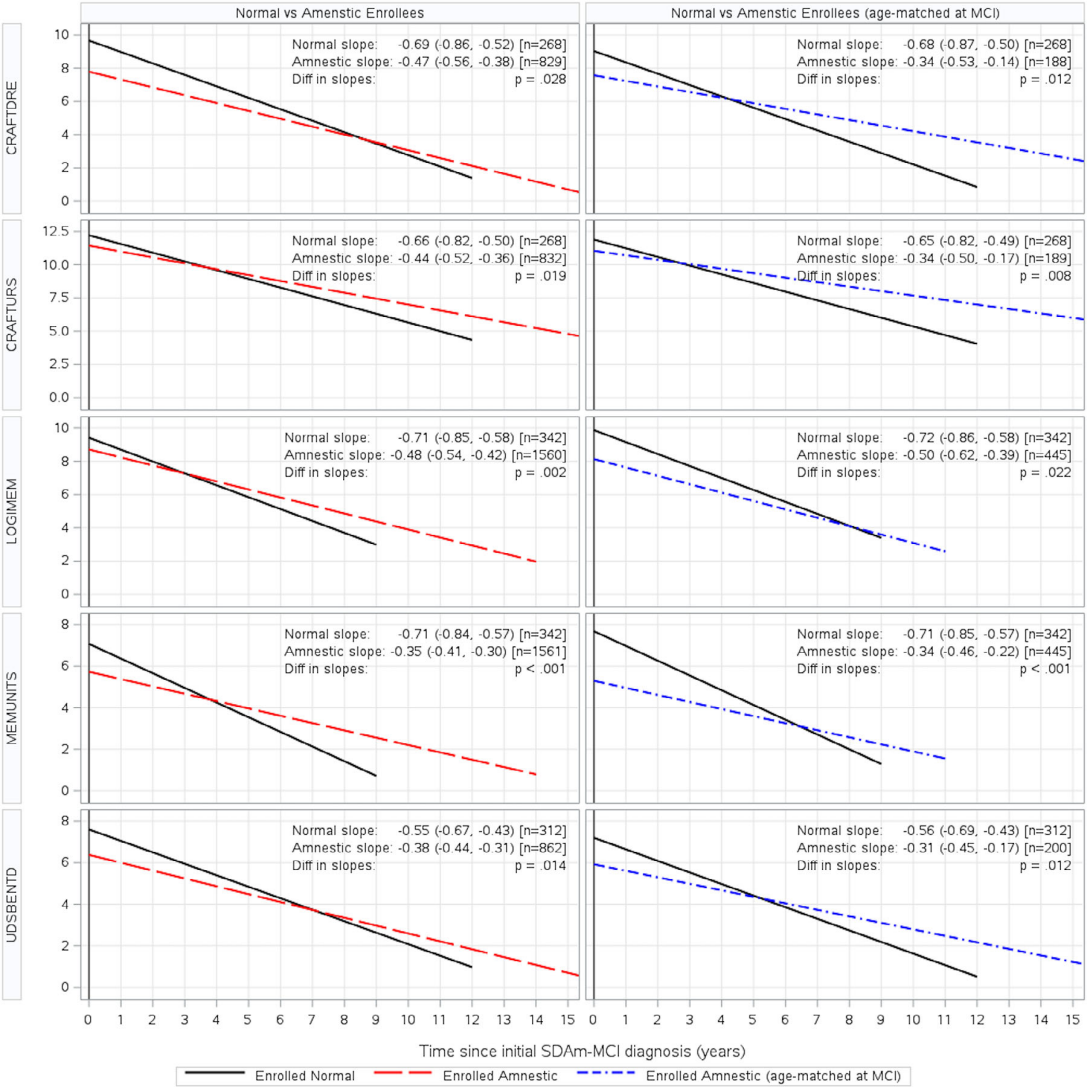
Comparison of memory measure trajectories during the symptomatic periods between Normal and Amnestic Enrollees, and Normal age‐matched Amnestic Enrollees. Time = 0 reflects time of SDAm‐MCI diagnosis. Time > 0 reflects time after diagnosis (symptomatic period). Depicted slopes represent fitted averages after adjusting for sex and age‐at‐initial SDAm‐MCI diagnosis. Linear slopes are graphically extended up to the observed minimum/maximum time from diagnosis among patients in respective cohorts. CRAFTDRE, Craft Story Delay; CRAFTURS, Craft Story Immediate; LOGIMEM, Logical Memory Immediate; MEMUNITS, Logical Memory Delay; UDSBENTD, Figure Recall; SDAm‐MCI, single domain amnestic mild cognitive impairment.

Both Normal and Amnestic Enrollee groups showed significant declining memory trajectory after their initial SDAm‐MCI diagnosis for all memory measures. However, Normal Enrollees had significantly higher rates of linear memory decline after diagnosis compared to Amnestic Enrollees on all memory measures, while adjusting for sex and age at initial SDAm‐MCI (Figure 2). The subset of Amnestic Enrollees who were 1:1 age matched at the time of initial SDAm‐MCI to Normal Enrollees showed similar differences between linear memory trajectories. All memory measures among Normal Enrollees declined at a significantly higher rate compared to the age‐matched Amnestic Enrollees (Figure 2).

## 4 DISCUSSION

Our results show memory measure raw scores (non‐age adjusted) were 17% to 39% less impaired for Normal Enrollees who converted to SDAm‐MCI during follow‐up, despite being an average of 7 years older at the time of diagnosis than those diagnosed with SDAm‐MCI at first research visit. This is consistent with our previous analysis in a clinical sample and a local aging research sample.^12^ Multiple memory measures showed this pattern, with the UDS C1 Logical Memory task showing the largest group difference. While memory measures distinguished the groups, overall global screening measures (MoCA and MMSE) did not. This supports our prior conclusion that longitudinal testing with neuropsychological measures allows for earlier diagnosis of SDAm‐MCI, the most common early symptomatic stage of AD.

These results also suggest that diagnostic transition from normal to MCI may be an inflection point at which time cognitive decline accelerates compared to pre‐symptomatic decline. However, the significant age difference at initial SDAm‐MCI epoch between the older Normal Enrollee group and the full sample of younger Amnestic Enrollee group is a limitation to this interpretation. We could not rule out that the acceleration of decline in the Normal Enrollee group is related to the compounded impact of more advanced age at the time of diagnosis. However, the same relative acceleration of decline in the Normal Enrollee group was present in the secondary analysis using the 1:1 age‐matched Amnestic Enrollee subgroup, suggesting that the accelerated relative decline after the time of diagnosis is not merely related to advanced age.

In our prior study, we also saw differences in non‐memory measures of cognition. In this sample, we did not see significant differences across other non‐memory domains in our planned analysis using the entire sample. However, in the 1:1 age‐matched subgroup analysis, we did see differences in other cognitive domains with the Amnestic Enrollee group showing lower scores in other domains at the time of diagnosis. This included Animal Fluency and Boston Naming, which were similarly significantly lower in our prior study,^12^ and the global screening measures (MMSE and MoCA). This is notable in that these changes did not reach a level of impairment to result in a diagnosis of multi‐domain impairment, which supports our assertion that by the time an individual with subjective cognitive concerns is evaluated, they are further along the MCI continuum with the beginnings of involvement of other cognitive domains in addition to memory than if an individual is followed and diagnosed longitudinally.

Observational follow‐up time is critical to characterizing these cognitive inflection points. This study faced a limitation in disproportionate amounts of symptomatic follow‐up time between study groups, with Amnestic Enrollees having longer follow‐up time, given that they were markedly younger at initial SDAm‐MCI diagnosis. Future studies prospectively evaluating longitudinal assessment and diagnosis within similar age bands are needed. Intervention with anti‐amyloid therapy may be especially relevant in this early diagnostic period, to slow or flatten the slope of decline over time, specifically when impairment is at its mildest clinically.

Additionally, this study faced common limitations associated with large cohort databases with data collections spanning long periods of time. Given the changes in neuropsychological battery used over time, this study was subject to missing data constraints. The results from this study were reported with observed sample size alongside estimates and figures to better frame interpretation given potential missing data constraints. In addition, we combined two UDS batteries, and the C2 battery has less longitudinal data than the C1 battery. Therefore, this is a limitation when evaluating trajectories within available data, as it is possible that the less commonly used measures (e.g., Craft Story Recall) lack similar potential to differentiate Normal and Amnestic Enrollees compared to other more commonly used measures (e.g., Logical Memory).

We analyzed SDAm‐MCI because this is the most common early presentation of typical AD. Therefore, interpretations of these results apply primarily to patients with amnestic‐driven cognitive decline, and it is possible that other AD subtypes may show different results on the neuropsychological tests as well as the cognitive screening tools. We hypothesize that following patients neuropsychologically during normal cognition will lead to earlier detection of cognitive changes with atypical presentations of AD or other neurodegenerative disease as well. However, we did not evaluate patients with initial diagnoses of non‐amnestic or multi‐domain decline. Given that amnestic MCI patients with evidence of amyloid pathology are a current target for disease‐modifying therapies, our findings are particularly relevant; however, future studies of other MCI groups are warranted, especially if disease‐modifying treatments for other neurodegenerative diseases are developed.

In conclusion, longitudinal neuropsychological evaluation is uniquely positioned to identify the earliest symptomatic time point of AD. The UDS neuropsychological battery is quite brief, typically requiring ≤ 30 minutes to administer^14^ compared to a more typical 2 to 4 hours for traditional neuropsychological evaluation. In addition, the UDS was developed to cover a range of cognitive domains in addition to memory.^16^ Such brief batteries are not universally appropriate for broad differential diagnosis in neuropsychology; however, for the purposes of targeted longitudinal screening, this type of brief battery may be appropriate in some settings, such as targeted cognitive screening clinics or primary care practices. Methodologies to increase access to neuropsychology with longitudinal evaluation protocols may help identify candidates for medical therapies and non‐pharmacological behavioral interventions as early as possible, maximizing the period of slowed cognitive decline, independent daily functioning, and high quality of life.

## CONFLICT OF INTEREST STATEMENT

Dona E. C. Locke: none, Blake Langlais: none, Dixie Woolston: none, Bryan K. Woodruff: none, Cynthia M. Stonnington: none, Leslie Baxter: none, Richard J. Caselli: Dr. Caselli received a consulting fee from Eli Lilly and consults for SPARK Neuroscience. Neither of these is related to this work. Author disclosures are available in the supporting information.

## CONSENT STATEMENT

This study used a de‐identified data set from the National Alzheimer's Coordinating Center so consent of subjects for this analysis was not necessary. All human subjects would have originally provided informed consent upon enrollment of their local NIA‐funded Alzheimer's Disease Research Center (ADRC), which would include consent to provide information to NACC.

## Supporting information



Supporting Information

Supporting Information
