# Supplementary material for: Longitudinal neuropsychological evaluation allows diagnosis of MCI with less severe memory change
Source: Alzheimers Dement. 2025 May 23;21(5):e70317. doi: 10.1002/alz.70317 (PMC12101964; doi:10.1002/alz.70317)
Supplement: Supplementary file 2 — Supporting Information [file ALZ-21-e70317-s002.docx]

Table S1. UDS battery comparison

| UDS C1[13] | UDS C2[14] |
| --- | --- |
| MMSE | MoCA |
| Logical Memory Immediate | Craft Story Immediate |
| Benson Complex Figure Copy | Benson Complex Figure Copy |
| Digit Span Forward | Number Span Forward |
| Digit Span Backward | Number Span Backward |
| Category Fluency (Animals, Vegetables) | Category Fluency (Animals, Vegetables) |
| Trail Making Test | Trail Making Test |
| Logical Memory Recall | Craft Story Recall |
| Benson Complex Figure Recall | Benson Complex Figure Recall |
| Boston Naming Test | Multilingual Naming Test |
| Verbal Fluency (F, L) | Verbal Fluency (F, L) |
